# Pain relief is associated with decreasing postural sway in patients with non-specific low back pain

**DOI:** 10.1186/1471-2474-13-39

**Published:** 2012-03-21

**Authors:** Alexander Ruhe, René Fejer, Bruce Walker

**Affiliations:** 1 Murdoch University, Praxis fuer Chiropraktik Wolfsburg, Wolfsburg, Germany; 2 Research Department, Spine Centre of Southern Denmark, Hospital Lillebaelt and University of Southern Denmark, Middelfart, Denmark; 3 School of Chiropractic and Sports Science, Murdoch University, Murdoch, Western Australia, Australia

## Abstract

**Background:**

Increased postural sway is well documented in patients suffering from non-specific low back pain, whereby a linear relationship between higher pain intensities and increasing postural sway has been described. No investigation has been conducted to evaluate whether this relationship is maintained if pain levels change in adults with non-specific low back pain.

**Methods:**

Thirty-eight patients with non-specific low back pain and a matching number of healthy controls were enrolled. Postural sway was measured by three identical static bipedal standing tasks of 90 sec duration with eyes closed in narrow stance on a firm surface. The perceived pain intensity was assessed by a numeric rating scale (NRS-11). The patients received three manual interventions (e.g. manipulation, mobilization or soft tissue techniques) at 3-4 day intervals, postural sway measures were obtained at each occasion.

**Results:**

A clinically relevant decrease of four NRS scores in associated with manual interventions correlated with a significant decrease in postural sway. In contrast, if no clinically relevant change in intensity occurred (≤ 1 level), postural sway remained similar compared to baseline. The postural sway measures obtained at follow-up sessions 2 and 3 associated with specific NRS level showed no significant differences compared to reference values for the same pain score.

**Conclusions:**

Alterations in self-reported pain intensities are closely related to changes in postural sway. The previously reported linear relationship between the two variables is maintained as pain levels change. Pain interference appears responsible for the altered sway in pain sufferers. This underlines the clinical use of sway measures as an objective monitoring tool during treatment or rehabilitation.

## Background

In a previous study we outlined that non-specific low back pain (NSLBP) intensity is correlated with the magnitude of postural sway [1]. This poses the question as to whether a) this relationship is maintained in case of pain reduction, in this case potentially associated with a manual therapeutic intervention and b) whether the resulting altered pain intensities correlate with similar center of pressure (COP) measures compared to participants that perceived identical pain intensities pre-intervention.

There is some evidence for "pain interference" as described by Crombez et al. [2] to be the predominate causative factor for the increased postural sway in pain sufferers [3,4]. Here, discharge from high-threshold nociceptive afferents interferes with spinal motor-pathways [5] as well as the motor cortex [6]. In addition it has been shown that pain may cause an increased pre-synaptic inhibition of muscle afferents [7] as well as affecting the central modulation of proprioceptive spindles of muscles [8], causing prolonged latencies by the decrease in muscle spindle feedback. These alterations may lead to decreased muscle control and result in increased postural sway.

Hodges advanced this concept to a new theory that proposes complementary, additive or competitive adaptations of the motor system during pain [9]. It has been shown that while the discharge rate of active motor units is reduced during experimental pain, the overall force output was maintained due to recruitment of additional, otherwise not active units [10]. These observations oppose the idea of a uniform "pain inhibition" of the motorneuron pool.

However, it has to kept in mind that for these experiments the motor recruitment pattern were investigated by EMG during voluntary, active movements. They do not necessarily reflect those employed involuntarily during static task conditions. Secondly, the nature of selective muscle actions observed on EMG (e.g. transversus abdominis [11]) may not necessarily correlate with postural sway.

Analgesic effects have been described for a variety of manual therapeutic interventions such as spinal manipulative therapy (SMT) [12,13], mobilization [12,14] or soft tissue techniques [15]. The mechanisms by which these techniques may produce hypoalgesia and restoration of biomechanical function are not well understood. There is, for example, still the unresolved controversy as to whether the mode of action behind the analgesic effects of manipulation is confined to spinal levels or involves a more complex, supraspinal mechanism [16].

The literature suggests a biomechanical effect of SMT on functional joint restrictions [17,18] to desensitize local nociceptors [18]. It involves the activation of mechanoreceptors in structures such as zygapophyseal joint capsules [19], spinal ligaments, intervertebral discs, the cutaneous receptors, muscle spindles and golgi tendon organs [19-22]. These afferent discharges may activate inhibitory interneurons to affect alpha motoneuron pools of the paraspinal musculature [23], breaking the pain-spasm-pain cycle.

As with manipulation, the clinical efficacy of mobilization procedures for pain reduction has been reported in the literature [12,14]. However, the physiologic mechanisms remain equally unclear, although mobilization has shown to elicit a profound but transient attenuation of motor neuron activity similar to that observed in spinal manipulations [24,25].

To our knowledge, this is the first study to investigate the association of altering pain levels and postural sway with a best practice experimental setup for COP measurements.

## Methods

### Subjects

The participants of this study were from a previously enrolled group of 77 NSLBP sufferers [1] that agreed to complete a course of three measurements and interventions. Based on their availability and willingness to participate we aimed at enrolling around 40 participants for both symptomatic and an aged-matched control group.

After oral and printed information had been given, the subjects consented to participate in this study, which was approved by the Murdoch University Human Research Ethics Committee (Approval 2010/173). The cut-off age for both controls and symptomatic individuals was 50 years as after that possible age-related sensory impairments may decrease postural stability [26-28]. Inclusion criteria for the symptomatic participants were NSLBP of any duration and the presence of pain ≥ 2 on the NRS-11 scale on the day of the postural sway recordings. We aimed at enrolling a broad spectrum of pain intensities between NRS scores 2 and 8. Participants were excluded if the pain went below the gluteal fold, there were positive nerve root findings, pronounced spinal deformities or previous traumatic injuries such as spinal fractures or whiplash associated disorders. No pain medication was allowed within 24 hrs prior to the recordings. Participants were also excluded if they were unable to perform the postural sway recording either due to pain or other reasons. For inclusion, patients had to consent to participate in three measurement sessions at 2-3 day intervals that were scheduled around their treatment appointments.

For the purpose of this study, healthy was defined as the absence of any self-reported neurological or musculoskeletal impairments, pain or disability for a minimum of 6 months prior to the time of evaluation. Specifically, individuals with a history of back pain within 6 months or previous injury to the neck or lower extremities, any known balance problems or the usage of medication associated with pain suppression or altered sensory perception were excluded. The physical examination of the control group must also have ruled out any back or extremity complaints or significant biomechanical impairments that might influence the measurements.

### Measurement equipment

The system used for this study was a Metitur Good Balance GB300^® ^CE (Metitur Oy, Finland). Signals were sampled at 100 Hz, amplified and converted from analogue to digital. High frequency noise was reduced by a low-pass filter with a cut-off frequency of 10 Hz.

### Procedures

The experimental setup was based on an earlier literature review where a best practice setup with regards to the reliability of COP data was published [29]. Accordingly, trials were conducted with eyes closed as the data obtained shows higher reliability than with eyes open. Mean velocity (mVel) was chosen as the main COP parameter as this has consistently shown to be both reliable [29] and discriminative for NSLBP [3].

The participants were asked to remove their shoes and stand upright on the forceplate with their eyes closed, the head erect and their arms hanging loosely by their sides. The foot position was narrow stance with toes and heels touching. Three successive trials of 90 sec duration each were conducted with a preceding 5 sec adaption period that was not recorded. The forceplate was calibrated prior to the recordings and further underwent an automatic calibration check before each trial.

Based on a physical examination, the participating NSLBP sufferers received a series of three non-specific therapeutic interventions at 2-3 day intervals consisting of a selection or a combination of all of the following: a) manipulation, b) mobilization, c) soft tissue techniques. The treatments were administered by two experienced chiropractors with 8 years of clinical practice each (TB and AS) and targeted the whole kinematic chain. Pain levels were assessed before each measurement session by an NRS-11 scale.

The practitioners performing the examination and delivering treatments were otherwise not involved in this study and blinded to the results of both the COP measures and the NRS-scores.

### Data analysis

To assess changes in postural sway velocity and NRS-scores, means, SDs and 95% CIs were calculated for all dependent variables (COP parameters) per session and NRS group. Independent samples *t*-test was performed to analyze differences in postural sway between pain intensity groups across the three measurements. The level of statistical significance was set at *p *≤ 0.05.

## Results

### Subjects

**Figure 1:**
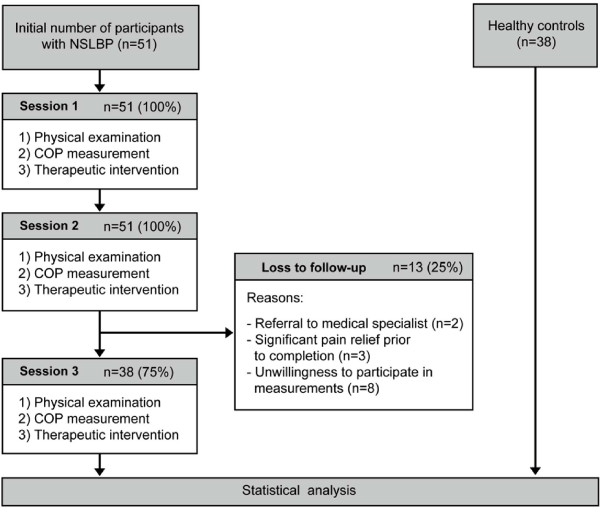
**Flowchart of procedures**.

Seventy-seven symptomatic participants enrolled in an earlier study [1] provided baseline data. From this group, 51 initially consented to participate in three measurements and to receive a series of manual interventions. Thirty-eight individuals (75%) suffering from NSLBP completed the full course. The following factors accounted for the loss to follow-up: Significant pain relief after less than three interventions (n = 3), unwillingness to participate in the COP measurements while continuing treatments (n = 8), discontinuation of chiropractic care and referral to medical specialist (n = 2). An identical group of healthy controls provided reference values for NRS scores 1-0. A comprehensive flowchart of procedures and participants is presented as Figure 1.

All participants were able to complete the trials without difficulty. The characteristics of the participants are shown in Table 1. Pain ranged from NRS 2-8 with n = 3 (NRS scores 2 and 3) to n = 10 (NRS score 8) participants per pain intensity group.

**Table 1:** Patient characteristics

	**NSLBP intervention group **(n = 38)	**Healthy controls **(n = 38)
Age (years)	39.8 ± 10.5	41.5 ± 5.5

Height (cm)	178.1 ± 8.4	176.9 ± 6.9

Weight (kg)	79.3 ± 12.4	76.9 ± 8.8

BMI	24.9 ± 3.1	24.5 ± 1.9

NRS-11 score at baseline	5.6 ± 2.0	N/A

Values are mean ± SD

### Pain intensity over the course of three therapeutic interventions

**Figure 2:**
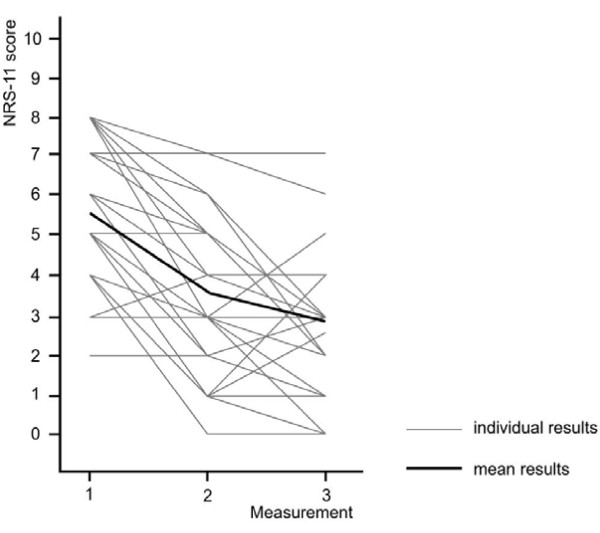
**Development of individual and mean NRS-scores over three measurements**. One grey line may indicate pain scores of several participants.

There was a significant decrease in pain intensity at measurement three (2.9 ± 1.6 (95% CI 2.2-3.3) compared to NRS 5.6 ± 2.0 (95% CI 4.9-6.2) at baseline (*p *≤ 0.001). Figure 2 shows the individual NRS scores as well as the average pain intensities. Where an increase in pain perception was reported at measurement 3 compared to the previous session, the final NRS-score was still lower compared to baseline.

### Relationship between pain intensity and postural sway

**Figure 3:**
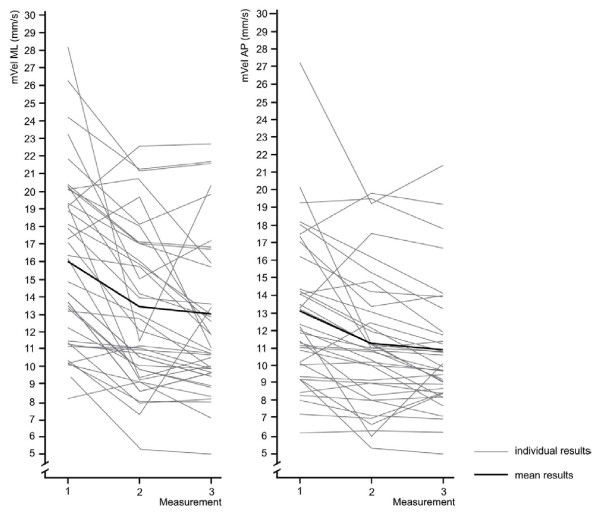
**Individual changes in mVel ML and AP over three measurements (n = 38)**.

All participants experienced pain relief over the course of the therapeutic interventions and all but two (2/38, 5%) exhibited lower associated postural sway velocities (Figure 3).

**Figure 4:**
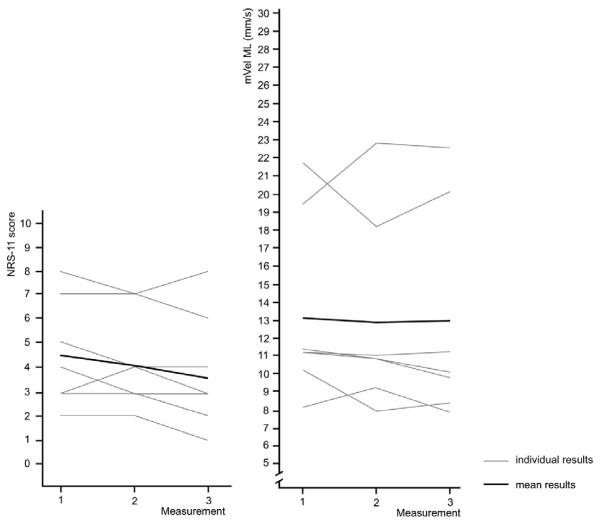
**Pain intensity and mVel ML for participants with a change in NRS scores of ≤ 1 over the course of three measurements (n = 7)**. One grey line may indicate pain scores of several participants.

**Figure 5:**
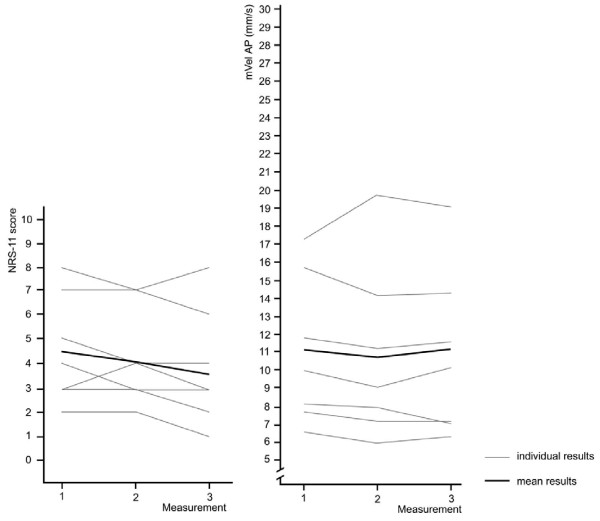
**Pain intensity and mVel AP for participants with a change in NRS scores of ≤ 1 over the course of three measurements (n = 7)**. One grey line may indicate pain scores of several participants.

The following figures show the relationship between pain intensity and postural sway for patients where the intervention did not result in pain reduction and the NRS scores changed less or equal to one score (n = 7, 18%). Overall pain intensity remained nearly constant between NRS 4.5 (baseline) and NRS 3.8 (measurement 3). At the same time, mVel ML remained at around 13 mm/s and mVel AP at around 11 mm/s. Postural sway associated with higher pain intensity at baseline shows greater variation while those at lower NRS scores remained very similar (Figure 4 and 5).

**Figure 6:**
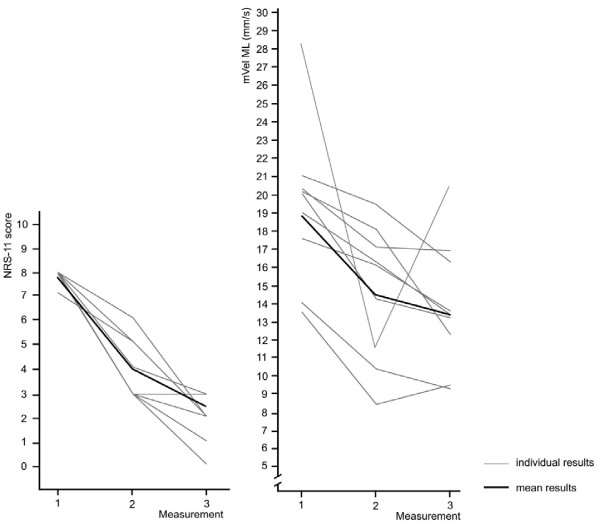
**Pain intensity and mVel ML for participants with a change in NRS scores of ≥ 4 over the course of three measurements (n = 9)**. One grey line may indicate pain scores of several participants.

**Figure 7:**
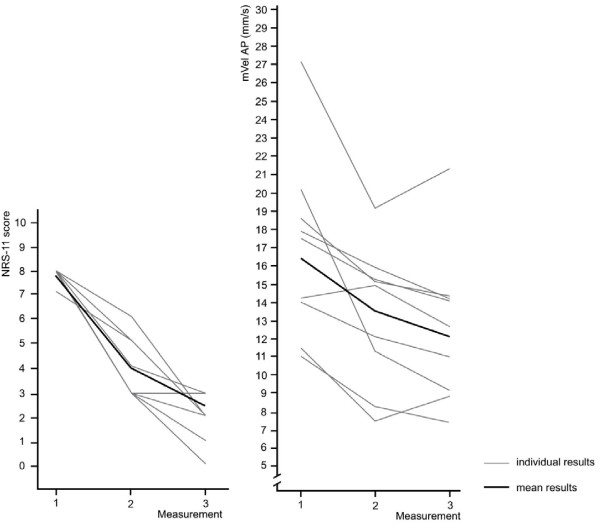
**Pain intensity and mVel AP for participants with a change in NRS scores of ≥ 4 over the course of three measurements (n = 9)**. One grey line may indicate pain scores of several participants.

Figures 6 and 7 demonstrate changes in sway velocity associated with a reduction in pain intensity of ≥ 4 NRS scores in 9 participants (24%). The mean NRS score changed from 7.8 at baseline to 2.7 at measurement 3. Mean sway velocity ML decreased from 18.8 mm/s to 13.7 mm/s and mVel AP from 16.5 mm/s to 12.5 mm/s at the same time. Despite reporting lower pain scores overall, one participant exhibited a greatly increased sway velocity at measurement 3 compared to session 2 and generally showed high variability in the COP results (Figure 6).

Figure 7 demonstrates the changes associated with decreasing NRS-scores for mean sway velocity in AP direction.

The results of the independent sample t-tests showed that with few exceptions there were no significant differences between a) the results of the first measurement (baseline) and the reference data and b) between the postural sway measured at session 2 and 3 compared to the reference values.

There were generally no significant differences in postural sway measures between those who experienced a certain pain intensity at one of the follow-up sessions compared to patients perceiving a similar pain at baseline. This was true for all included COP parameters (Figure 2-3).

As a general trend, higher pain intensities at session 2 and 3 that were most likely reported by patients with the highest NRS scores at baseline (NRS score 8) were associated with greater variability in postural sway compared to those associated with lower pain scores. As in contrast to medium and low pain intensities sway associated with NRS scores 6-8 showed mostly non-overlapping 95% CIs, observing sway data for these scores across the trials may offer particularly valuable insights. All patients reporting these NRS levels experienced pain relief and therefore no data sets were included twice for the same pain score. The respective values appear shaded in gray on the following tables (Tables 2 and 3).

**Table 2:** Changes in postural sway velocity ML across three repeated measurements at 2-3 day intervals

NRS score	Reference values	Measurement 1			Measurement 2			Measurement 3		
		**n**	**mVel ML**	**p-value**	**n**	**mVel ML**	**p-value**	**n**	**mVel ML**	**p-value**

**8**	**21.2 (20.0-22.5)**	10	20.4 (18.8-22.0)	0.91	0	-	-	0	-	-

**7**	**18.6 (17.7-19.4)**	5	19.3 (17.5-21.2)	0.40	3	19.8 (16.9-22.7)	0.37	0	-	-

**6**	**15.6 (14.7-16.6)**	5	15.6 (12.9-18.2)	0.93	4	19.4 (15.7-23.1)	**0.04**	2	22.4 (20.6-24.2)	**≤ 0.001**

**5**	**13.9 (12.6-15.2)**	7	14.7 (12.7-16.7)	0.47	5	14.5 (12.2-17.6)	0.45	2	17.5 (9.5-25.4)	0.19

**4**	**13.1 (12.0-14.2)**	5	11.5 (10.0-13.0)	0.09	5	16.1 (13.0-19.2)	**0.04**	5	12.8 (11.5-14.0)	0.68

**3**	**12.4 (11.4-13.4)**	3	12.0 (10.4-13.7)	0.72	14	12.1 (10.7-13.5)	0.70	10	12.8 (11.0-14.5)	0.71

**2**	**11.5 (10.7-12.3)**	3	12.1 (10.3-13.9)	0.47	5	11.0 (10.2-11-9)	0.48	13	11.8 (10.7-13.0)	0.59

**1-0**	**11.0 (10.5-11.7)**	0	-	-	2	12.5 (11.2-13.8)	0.16	6	12.0 (11.0-13.1)	0.19

Reference values are based on 11 participants per NRS score.

Values are mean and (95% CI)

**Table 3:** Changes in postural sway velocity AP across three repeated measurements at 2-3 day intervals

NRS score	Reference values	Measurement 1			Measurement 2			Measurement 3		
		**n**	**mVel ML**	**p-value**	**n**	**mVel ML**	**p-value**	**n**	**mVel ML**	**p-value**

**8**	**18.4 (16.8-20.0)**	10	17.5 (15.4-19.6)	0.71	0	-	-	0	-	-

**7**	**15.8 (14.7-16.9)**	5	14.9 (13.4-16.5)	0.84	3	15.1 (12.3-17.8)	0.60	0	-	-

**6**	**13.0 (12.2-13.7)**	5	14.6 (12.2-16.9)	0.11	4	15.1 (11.4-18.8)	0.08	2	16.9 (14.5-19.3)	**0.006**

**5**	**12.9 (11.3-14.4)**	7	11.0 (9.2-12.7)	0.10	5	11.8 (9.9-13.7)	0.43	2	17.3 (8.5-26.2)	0.13

**4**	**11.2 (10.1-12.4)**	5	9.8 (8.6-11.0)	0.11	5	15.9 (12.8-18.9)	**0.005**	5	10.8 (9.0-12.6)	0.67

**3**	**11.1 (10.1-12.2)**	3	11.2 (8.7-13.7)	0.95	14	10.1 (9.1-11.1)	0.15	10	11.5 (9.7-13.2)	0.73

**2**	**9.8 (9.0-10.6)**	3	10.0 (8.9-11.2)	0.76	5	9.3 (7.3-11.4)	0.61	13	10.5 (9.7-11.3)	0.21

**1-0**	**8.9 (8.5-9.5)**	0	-	-	2	10.2 (9.6-10.8)	0.79	6	9.4 (8.1-10.7)	0.43

Reference values are based on 11 participants per NRS score.

Values are mean and (95% CI)

## Discussion

This study aimed to investigate alterations in postural sway associated with decreasing pain intensities. As an observational study with no control group or randomization of patients, it was neither intended nor designed to investigate any effect of manual therapies on non-specific low back pain.

While the reduction in pain followed a course of manual interventions, placebo, analgesic medication or natural history may have elicited similar results with regards to the associated sway alterations. The study design did not intend or allow to assess or quantify any potential additional biomechanical benefit of the therapeutic intervention on postural stability.

During the course of the three measurements, a dropout rate of 25% (13/51) occurred at session three while the groups at measurement 2 remained complete. As the data of these participants was completely removed from the study, no further statistical adjustment such as "intention-to-treat" analysis was deemed necessary. Although for a longitudinal observational study incomplete data sets are not necessarily excluded, their removal was deemed appropriate as individual results are followed over the course of the three sessions. The inclusion of incomplete sway data may adversely affect group means per measurement due to inter-subject variability and thereby alter the interpretation of the results.

Irrespective of the unclear underlying mechanism, the observed decrease in pain intensity over three measurements exceeded two points on an NRS score and is therefore considered a clinically relevant change [30-32]. The study design did not set out to distinguish whether one treatment was associated with a more significant decrease in pain compared to others and therefore this cannot be commented on.

Previously we were able to demonstrate a linear relationship between COP sway and NRS scores in NSLBP patients [1]. The trend observed in this study further strengthens the impression that this close association between postural sway and pain intensity also exists if the original pain NRS-scores change.

The pain reduction occurred following a series of non-specific therapeutic interventions. As mentioned, any contribution of intervention, placebo effects or pain remission due to natural cause remains unclear. As a general trend, both group means and individual COP measurements indicate that a decrease in postural sway was observed if NRS scores also decreased. If this was not the case, the postural sway remained similar (Figures 6 and 7).

While learning effects cannot be excluded as an explanation for altered postural sway at follow-up, this appears less likely as similar effects would be excepted for those patients where no decrease in pain occurred. However, no such effect was observed.

Some studies do not support our observations as no decreasing postural sway was observed following pain reduction [33-35]. This may be attributed to the fact that patients with neurological impairments were enrolled where demyelination probably inhibited full recovery within the follow-up period [34,35]. Secondly, these studies employed prolonged follow-up periods of 3 [34] and 6 months [35], while we investigated short-term changes over the course of around 2 weeks.

The chronic low back pain patients without neurological symptoms enrolled by Mehling et al. [33] experienced only a minimal average pain decrease (VAS 5.15 ± 2.0 at baseline compared to VAS 4.37 ± 2.36 post-intervention). This is not considered a clinically significant improvement and sway data published in an earlier study did also not identify a significant change in postural sway between those pain scores [1].

In addition, the results may have been affected by high inter-subject variability associated with the small sample sizes or single measurements of short duration [29].

The results of our study warrant caution in interpretation. First of all, pain perception is multifactorial [36] and in addition to functional impairments, psychological aspects may play an important role. This was not assessed for and can therefore not conclusions can be reached regarding their implications. It is further possible that both intra- and inter-subject variability in postural sway is masked when calculating means and therefore difficult to interpret.

In addition, the data shows quite wide variations in postural sway velocity likely due to the low sample sizes, particularly at medium pain intensities. When groups consisted of larger patient numbers, generally no significant sway differences were observed compared to other patients experiencing similar pain at baseline. The results from this study suggest that each group should consist of around 10 participants for further analyses. Considering a dropout rate of around 25%, about 14 participants should therefore be enrolled. However, with regards to assessing changes in sway or pain intensity at the follow up recordings, sample size calculations are unable to take this into account as the number of patients that did or did not show alterations in the variable of interest cannot be predicted.

At first sight, these results are quite interesting as a larger inherent variability would have be expected. On the other hand, it is consistent with the subjective nature of pain perception. If a group of individuals receives an identical painful stimulus, a certain variation in pain perception will occur as a result [37]. However, this study suggests that similar postural sway responses occur in those patients reporting the same NRS-score. Secondly, the overlapping 95% CIs for all COP parameters observed between NRS scores particularly at lower NRS scores (Chapters 9 and 10) make results within the same range more probable.

The results further suggest that the presence of pain may be responsible for alterations in postural sway [11] rather than changes/alterations in proprioceptive information caused by chronic damage to sensory tissues in the neck. Even considering neural plasticity, any reversal of such alterations appears unlikely within the 2-3 day period between measurements.

Further investigations with larger sample sizes are needed to confirm the observed trend for all NRS-scores. Another approach would be to investigate whether this can also be observed when observing natural remission. Also, studies employing analgesics are indicated to further assess the role of direct pain relief compared to the biomechanical, functional approach applied here. Comparing such observations may also indicate whether there are additional benefits associated with manual therapies such as spinal manipulation.

### Clinical applications

Although the results have to be interpreted with some caution, the COP measurement protocol used in this study may be suitable as an objective outcome measure for clinical monitoring purposes. This in turn also suggests that pain assessment by NRS-11 may be equally objective, thereby limiting the clinical use of COP measures for this specific purpose.

As previously described, it has been demonstrated that elderly fallers show significantly increased postural sway compared to non-fallers [38-40]. There is also evidence that higher COP sway is associated with a higher risk of falling [41] and sustaining injuries as a consequence, although this is subject to debate [42,43]. Consequently, if such individuals are additionally suffering from pain, this may further increase the risk of falling in addition to any age-related or pathological changes in postural stability. As this study shows lower sway to be associated with decreasing pain intensities, this underlines the importance of pain control particularly in this population to reduce COP sway and increase postural stability.

### Limitations

There are various limitations to this study. The issues associated with small sample sizes became even more pronounced by the fact that the number of patients per NRS score varied considerably as pain levels changed. Some NRS groups consisted of only n = 2, as seen particularly at higher pain intensities as pain levels decreased over the course of the measurements. This rendered a meaningful statistical analysis difficult. On the other hand, other pain groups grew to n = 14 as a result, which strengthened the conclusions drawn from this data.

In addition, the study design did not allow to determine whether decreasing pain scores alone was responsible for the decreasing postural sway or whether the manual intervention added an additional benefit by increasing biomechanical function. Based on the available literature, however, the latter appears unlikely to exhibit any significant effect (Chapter 5). Furthermore, the cut-off age of 50 years does not allow to extend the results to a geriatric population as the decreased pain perception in this age group [44] may not lead to similar postural responses. The same accounts for adolescents and children.

## Conclusions

Irrespective of the subjective nature of pain perception and the unclear causative factors, the results of this study suggest that there is a close association between the COP parameters and perceived pain levels even if pain levels change. Although the results have to be interpreted with some caution, the COP measurement protocol used in this study may be suitable as an objective outcome measure for clinical monitoring purposes. However, this in turn also suggests that pain assessment by NRS-11 may be equally objective, thereby potentially limiting the clinical use of COP measures for this specific purpose.

## Competing interests

The authors declare that they have no competing interests.

## Authors' contributions

AR carried out the COP measurements, conducted the statistical analysis and drafted the manuscript. RF and BW participated in the study design and assisted in drafting the manuscript. All authors read and approved the final manuscript.

## Pre-publication history

The pre-publication history for this paper can be accessed here:

http://www.biomedcentral.com/1471-2474/13/39/prepub
